# A postsurgical prognostic nomogram for patients with lymph node positive rectosigmoid junction adenocarcinoma

**DOI:** 10.1186/s12876-023-02810-7

**Published:** 2023-05-18

**Authors:** Wu Yanlong, Wu Yunxiao, Wang Yibing

**Affiliations:** 1 Department of Medical Records, The Second Affiliated Hospital of Nanchang University, Nanchang, China; 2 Department of Neurosurgery, The First Affiliated Hospital of Nanchang University, Nanchang, China; 3 Department of Emergency, The Second Affiliated Hospital of Nanchang University, Nanchang, China

**Keywords:** Nomogram, Postsurgical prognostic, Rectosigmoid junction adenocarcinoma

## Abstract

**Objective:**

The definition of rectosigmoid junction (RSJ) is still in debate. The treatment and prognosis of patients with rectosigmoid junction cancer (RSJC) and positive lymph nodes (PLN-RSJCs) are mostly based on the American Joint Committee on Cancer (AJCC) staging system. Our study aims to assist clinicians in creating a more intuitive and accurate nomogram model for PLN-RSJCs for the prediction of patient overall survival (OS) after surgery.

**Methods:**

Based on the Surveillance, Epidemiology, and End Results (SEER) database, we extracted 3384 patients with PLN-RSJCs and randomly divided them into development (*n* = 2344) and validation (*n* = 1004) cohorts at a ratio of 7:3. Using univariate and multivariate COX regression analysis, we identified independent risk factors associated with OS in PLN-RSJCs in the development cohort, which were further used to establish a nomogram model. To verify the accuracy of the model, the concordance index (C-index), receiver operating characteristic (ROC) curves, calibration curves, and an internal validation cohort have been employed. Decision curve analysis (DCA) was used to assess the clinical applicability and benefits of the generated model. Survival curves of the low- and high-risk groups were calculated using the Kaplan–Meier method together with the log-rank test.

**Results:**

Age, marital, chemotherapy, AJCC stage, T and N stage of TNM system, tumor size, and regional lymph nodes were selected as independent risk factors and included in the nomogram model. The C-index of this nomogram in the development (0.751;0.737–0.765) and validation cohorts (0.750;0.764–0.736) were more significant than that of the AJCC 7th staging system (0.681; 0.665–0.697). The ROC curve with the calculated area under the curve (AUC) in the development cohort was 0.845,0.808 and 0.800 for 1-year, 3-year and 5-year OS, AUC in the validation cohort was 0.815,0.833 and 0.814 for 1-year, 3-year and 5-year, respectively. The calibration plots of both cohorts for 1-year,3-year and 5-year OS all demonstrated good agreement between actual clinical observations and predicted outcomes. In the development cohort, the DCA showed that the nomogram prediction model is more advantageous for clinical application than the AJCC 7th staging system. Kaplan–Meier curves in the low and high groups showed significant difference in patient OS.

**Conclusions:**

We established an accurate nomogram model for PLN-RSJCs, intended to support clinicians in the treatment and follow-up of patients.

## Introduction

According to the 2022 Cancer Statistics Report, about 609,360 people died from cancer in the United States. Colorectal cancer is the second leading cause of death, after lung cancer [1]. Roughly, 140 people die of colorectal cancer every day in the United States. [1]. The rectosigmoid junction (RSJ) is the connection between the sigmoid colon and the upper rectum [2, 3]. However, its exact definition is still debatable [4]. Anatomically, the RSJ is the tissue at the end of the sigmoid colon [5], yet some experts still prefer to attribute RSJ to the rectum, as it shares blood vessels and other support with the upper rectum [6]. In general, RSJ is usually attributed to the upper rectum, according to the AJCC staging system [7]. Due to the controversial location of RSJ and the relative rarity RSJ adenocarcinoma (RSJC), relevant clinical data is scarce, making its treatment difficult and patient prognosis hard to predict. The purpose of our study is to generate a reliable RSJC risk prediction model, that supports the prediction of the prognosis of RSJC patients with positive lymph nodes (PLN-RSJCs) after surgery.

The nomogram model is a digital simulation tool widely used in clinical research and its results can be recognized by the public [8–10]. By scoring each single predictor, total scores can be obtained in different stages of the disease, enabling the prognosis prediction in a more intuitive manner [11]. A model for the perioperative treatment of locally advanced rectosigmoid colon has been previously established by Chao Zhang and colleagues [12]. This study showed that the current perioperative neoadjuvant therapy for rectal cancer is different from that of RSJC, and proved that lymph node positivity is an important factor in the selection of treatment for patients with locally advanced RSJC [12].

The Surveillance, Epidemiology, and End Results (SEER) database, established by the National Cancer Institute (NCI), collects data on cancer diagnosis, treatment, and survival in the U.S. population. On average, around 400,000 cancer cases are collected each year, providing researchers with a large amount of retrospective data. The SEER medical database has been use in clinical research aiming at improving the outcome of colorectal cancer patients [13].

To the best of our knowledge, this is the first study to establish a prognostic model for PLN-RSJCs after surgery.

## Methods

### Patient selection and data processing

**Fig.1:**
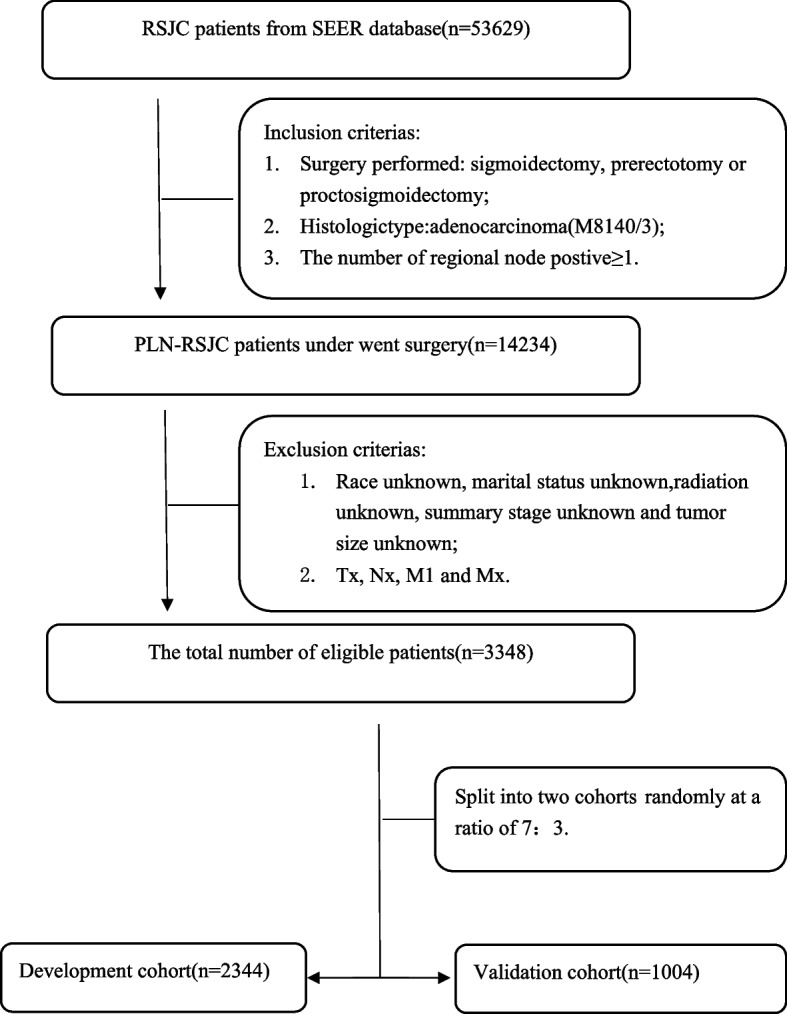
Data screening flow chart

Data from PLN-RSJCs patients were selected from the SEER database using the SEER*Stat software version 8.4.0 (www.seer.cancer.gov/seerstat). Patients were selected based on the third edition of the International Classification of Diseases for Oncology (ICD-O-3). Inclusion criteria comprised: surgery performed, including sigmoidectomy, prerectotomy or proctosigmoidectomy; histologic type: adenocarcinoma M8140/3(morphological coding for adenocarcinoma diagnosis); and positive regional nodes ≥ 1. Exclusion criteria were as follows: race unknown, marital status unknown; radiation unknown; summary stage unknown; tumor size unknown; Tx; Nx; M1 and Mx. According to the above criteria, we included 3348 eligible PLN-RSJCs patients in our retrospective study. The data were then randomly split into development (n = 2344) and validation (n = 1004) cohort at a ratio of 7:3 (Fig. 1).

### Variables defined

The variables in the selected cohort included: age, sex, race, marital, AJCC stage (7th), T stage, N stage, summary stage, radiotherapy, chemotherapy, tumor size, examined and positive regional nodes. To facilitate the analysis, several continuous variables (age, tumor size, examined regional nodes, and positive regional nodes positive) were transformed into categorical variables using the X-Tile software (Yale School of Medicine, New Haven, CT, USA), which calculates the best cutoff of the continuous variables: age (< 45, 45–64, 65–84, and ≥ 85), tumor size (≤ 3.7 cm, 3.8–5.6 cm, ≥ 5.7 cm), examined regional nodes (1–12, 13–16, ≥ 17), positive regional nodes (1–3, 4–8, ≥ 9). Sex was divided into female and male, and race included White, Black, Asian or pacific islander, and American Indian or Alaska native. We defined marital as single, married, divorced (or separated), widowed and unmarried or domestic partner.

### Statistical analysis

For the analysis of continuous and categorical variables in both development and validation cohort, the T and Chi-square tests were used. Univariate COX regression was used to extract the potential significant predictors in the development cohort. Predictors with *p* value lower than 0.05 were included in the multivariate COX proportional risk regression model. The independent prognostic factors with p lower 0.05 in the multivariate COX model were incorporated into the nomogram model, to establish a visual prediction model to evaluate the 1-year, 3-year and 5-year survival rates of patients. Hazard ratios and 95% confidence intervals are presented for all results.

To assess the performance of the model, we used concordance index (C-index) and receiver operating characteristic (ROC) curves with the calculated area under the curve (AUC). Furthermore, calibration plots were used to evaluate the consistency of predicted and actual survival time at 1-year, 3-year and 5-year points in time. The clinical applicability and benefits of the prediction model were estimated using decision curve analyses (DCA). Finally, the development cohort was divided in two risk groups, based on the respective total points. The Kaplan–Meier method, combined with the log-rank test, was applied to analyze the differences in the OS between the low- and high-risk groups. Statistical analysis was performed with the SPSS 22.0 (IBM Corp, Armonk, NY) and R version 4.2.0 software.

## Results

### Characterization of eligible patients

The PLN-RSJCs patients (*n* = 3348) from the SEER database were randomly divided at a ratio of 7:3 into development (*n* = 2344) and validation (*n* = 1004) cohort. A summary of the demographic and clinicopathological characteristics of the study population is presented in Table 1. No significant differences were observed in any of the considered parameters between the two cohorts.

**Table 1:** Demographics and clinical characteristics of eligible patients

Characteristics	Total cohort N(%)	Development cohort N (%)	Validation cohort N (%)	*P*-value
**Number of patients**	3348	2344(70%)	1004(30%)	
**Age**				0.732
< 45	330(9.9%)	236(10.1%)	94(9.3%)	
45–64	1613(48.1%)	1128(48.1%)	485(48.5%)	
65–84	1234(36.9)	866(36.9)	368(36.7%)	
≥ 85	171(5.1%)	114(5.9%)	57(5.7%)	
**Sex**				0.396
Female	1480(44.2%)	1025(43.7%)	455(45.3%)	
Male	1868(55.8%)	1319(56.3%)	549(54.7%)	
**Race**				0.343
White	2623(78.3%)	1852(79.0%)	771(76.8%)	
Black	292(8.7%)	202(8.6%)	90(9.0%)	
Asian or Pacific Islander	412(12.3%)	274(11.8%)	138(13.7%)	
American Indian/Alaska Native	21(0.7%)	16(0.7%)	5(0.5%)	
**Marital**				0.513
Single	651(19.5%)	471(20.1%)	180(17.9%)	
Married	1923(57.4%)	1330(56.7%)	593(59.1%)	
Divorced or Separated	355(10.6%)	255(10.9%)	100(10%)	
Widowed	404(12.1%)	278(11.9%)	126(12.5%)	
Unmarried or Domestic Partner	15(0.4%)	10(0.4%)	5(0.5%)	
**Radiation**				0.559
No	2586(77.2%)	1817(77.5%)	769(76.6%)	
Yes	762(22.8%)	527(22.5%)	235(23.4%)	
**Chemotherapy**				0.204
No	968(28.9%)	693(2936%)	275(27.4%)	
Yes	2380(71.1)	1651(70.4%)	729(72.6%)	
**Summary_stage**				0.595
Regional	2551(76.2%)	1780(75.9%)	771(76.8%)	
Distant	797(23.8%)	564(24.1%)	233(23.2%)	
**AJCC_Stage**				0.086
IIIA	330(9.9%)	228(9.7%)	102(10.2)	
IIIB	1653(49.3%)	1168(49.8%)	485(48.3%)	
IIIC	625(18.7%)	417(17.8%)	208(20.7%)	
IVA	489(14.6%)	340(14.5%)	149(14.8%)	
IVB	251(7.5%)	191(8.2%)	60(6.0%)	
**T**				0.918
T1	66(2.0%)	47(2.0%)	19(1.9%)	
T2	351(10.5%)	244(10.4%)	107(10.7%)	
T3	2210(66.0%)	1555(66.3%)	655(65.2%)	
T4a	497(14.8%)	347(14.8%)	150(15.0%)	
T4b	224(6.7%)	151(6.5%)	73(7.2%)	
**N**				0.726
N1a	921(27.5%)	657(28.0%)	264(26.3%)	
N1b	986(29.5%)	695(29.7%)	291(29.0%)	
N1c	5(0.1%)	3(0.1%)	2(0.2%)	
N2a	726(21.7%)	498(21.3%)	228(22.7%)	
N2b	710(21.2%)	491(20.9%)	219(21.8%)	
**Tumor_size**				0.924
≤ 3.7 cm	849(25.4%)	590(25.2%)	259(25.8%)	
3.8–5.6 cm	1579(47.1%)	1107(47.2%)	472(47.0%)	
≥ 5.7 cm	920(27.5%)	647(27.6%)	273(27.2%)	
**Regional_nodes_examined**				0.640
1–12	686(20.5%)	489(21.9%)	197(19.6%)	
13–16	842(25.1%)	592(25.3%)	250(24.9%)	
≥ 17	1820(56.4%)	1263(53.8%)	557(55.5%)	
**Regional_nodes_positive**				0.345
1–3	1913(57.1%)	1358(57.9%)	555(55.3%)	
4–8	976(29.2%)	668(28.5%)	308(30.7%)	
≤ 9	459(13.7%)	318(13.6%)	141(14.0%)	

### Prognostic factors in development cohort

Univariate and multivariate analyses were applied to extract independent prognostic factors from the development cohort. The results revealed that age, marital, chemotherapy, AJCC stage, T stage, N stage, tumor size, number of examined regional nodes were independent prognostic factors for PLN-RSJCs patients (Table 2).

**Table 2:** Univariate and multivariate regression analyses for OS

Characteristics	Univariate analysis HR (95%CI)	*P*-value	Multivariate analysis HR (95%CI)	*P*-value
**Age**				
< 45	Ref		Ref	
45–64	0.978 (0.792–1.210)	0.844	1.124(0.906–1.394)	0.287
65–84	1.541(1.247–1.903)	< 0.001*	1.767(1.416–2.205)	< 0.001*
> = 85	2.975(2.256–3.925)	< 0.001*	2.998(2.193–4.099)	< 0.001*
**Sex**				
Female	Ref			
Male	1.007(0.898–1.129)	0.906		
**Race**				
White	Ref		Ref	
Black	1.392(1.153–1.681)	< 0.001*	1.188(0.980–1.440)	0.079
Asian or Pacific Islander	0.978(0.814–1.174)	0.810	1.008(0.837–1.212)	0.937
American Indian/Alaska Native	0.552(0.229–1.330)	0.185	0.510(0.210–1.240)	0.138
**Marital**				
Single	Ref		Ref	
Married	0.712(0.615–0.824)	< 0.001*	0.741(0.637–0.862)	< 0.001*
Divorced or Separated	1.111(0.908–1.357)	0.309	1.138(0.925–1.398)	0.220
Widowed	1.370(1.137–1.652)	< 0.001*	0.854(0.694–1.051)	0.136
Unmarried or Domestic Partner	1.099(0.454–2.664)	0.834	1.592(0.652–3.883)	0.307
**Radiation**				
No	Ref		Ref	
Yes	0.739(0.641–0.851)	< 0.001*	1.028(0.882–1.199)	0.721
**Chemotherapy**				
No	Ref		Ref	
Yes	0.498(0.443–0.560)	< 0.001*	0.468(0.408–0.537)	< 0.001*
**Summary_stage**				
Regional	Ref		Ref	
Distant	3.356(2.979–3.781)	< 0.001*	1.122(0.673–1.871)	0.689
**AJCC_Stage**				
IIIA	Ref		Ref	
IIIB	2.116(1.582–2.831)	< 0.001*	1.007(0.596–1.703)	0.979
IIIC	3.112(2.289–4.229)	< 0.001*	1.080(0.606–1.912)	0.793
IVA	6.139(4.529–8.320)	< 0.001*	2.816(1.322–5.996)	0.007*
IVB	10.779(7.854–14.792)	< 0.001*	4.024(1.888–8.576)	< 0.001*
**T**				
T1	Ref		Ref	
T2	1.012(0.532–1.925)	0.971	0.971(0.507–1.861)	0.929
T3	2.440(1.346–4.424)	0.003*	1.830(0.871–3.847)	0.111
T4a	4.553(2.487–8.334)	< 0.001*	2.832(1.333–6.017)	0.007*
T4b	5.213(2.806–9.683)	< 0.001*	2.393(1.100–5.205)	0.028*
**N**				
N1a	Ref		Ref	
N1b	1.272(1.086–1.492)	0.003*	1.229(1.046–1.443)	0.012*
N1c	2.212(0.709–6.903)	0.171	4.400(1.329–14.564)	0.015*
N2a	1.368(1.153–1.624)	< 0.001*	2.021(0.579–7.050)	0.270
N2b	2.140(1.820–2.516)	< 0.001*	2.563(0.733–8.964)	0.141
**Tumor_size**				
≤ 3.7 cm	Ref		Ref	
3.8–5.6 cm	1.268(1.094–1.470)	0.002*	1.008(0.864–1.175)	0.919
≥ 5.7 cm	1.711(1.461–2.003)	< 0.001*	1.194(1.007–1.416)	0.040*
**Regional_nodes_examined**				
1–12	Ref		Ref	
13–16	0.703(0.599–0.825)	< 0.001*	0.742(0.630–0.874)	< 0.001*
≥ 17	0.652(0.568–0.748)	< 0.001*	0.590(0.509–0.685)	< 0.001*
**Regional_nodes_positive**				
1–3	Ref		Ref	
4–8	2.061(1.131–1.469)	< 0.001*	0.620(0.180–2.140)	0.450
≥ 9	2.061(1.765–2.407)	< 0.001*	0.844(0.243–2.930)	0.789

^*^Statistical significance

### Nomogram construction

**Fig. 2:**
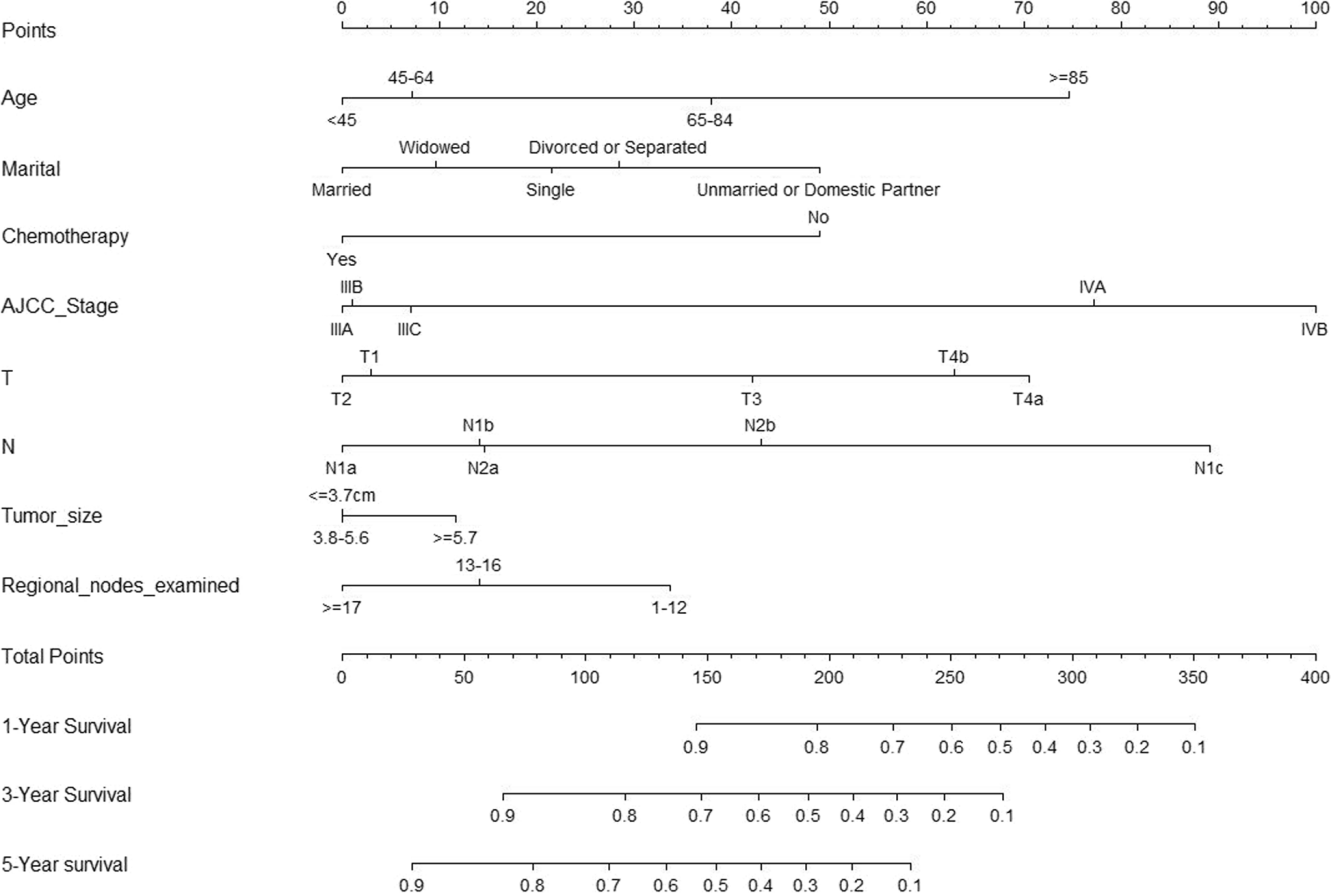
Nomogram for OS prediction in PLN-RSJCs

Based on the previous results of multivariate analysis in the development cohort, we integrated these independent prognostic factors to establish a nomogram model for OS prediction in PLN-RSJCs (Fig. 2). Each variable in the nomogram was assigned a corresponding score from 0 to 100, based on the contribution to the nomogram model (Table 3). Therefore, for each patient we obtained a total number of points by adding the scores in each subgroup. By this method, we were able to predict the possibility of 1-year, 3 year, and 5-year OS. Higher scores were negatively associated with patient prognosis.

**Table 3:** Nomogram scoring system

Variables	points	Variables	points
**Age**		**Marital**	
< 45	0	Single	21
45–64	7	Married	0
65–84	38	Divorced or Separated	28
≥ 85	75	Widowed	10
		Unmarried or Domestic Partner	49
**Chemotherapy**		**AJCC Stage**	
No	49	IIIA	0
Yes	0	IIIB	1
		IIIC	7
		IVA	77
		IVB	100
**T**		**N**	
T1	3	N1a	0
T2	0	N1b	14
T3	42	N1c	89
T4a	71	N2a	15
T4b	63	N2b	43
**Tumor size**		**Regional nodes examined**	
≤ 3.7 cm	0	1–12	34
3.8 cm-5.6 cm	0	13–16	14
≥ 5.7 cm	12	≥ 17	0
**1-Year Survival**		**3-Year Survival**	
0.9	145	0.9	66
0.8	195	0.8	116
0.7	226	0.7	147
0.6	250	0.6	171
0.5	271	0.5	191
0.4	289	0.4	210
0.3	307	0.3	228
0.2	327	0.2	247
0.1	350	0.1	271
**5-Year survival**		**5-Year survival**	
0.9	28	0.4	172
0.8	78	0.3	190
0.7	109	0.2	210
0.6	133	0.1	233
0.5	154		

### Validation of the nomogram

**Fig. 3:**
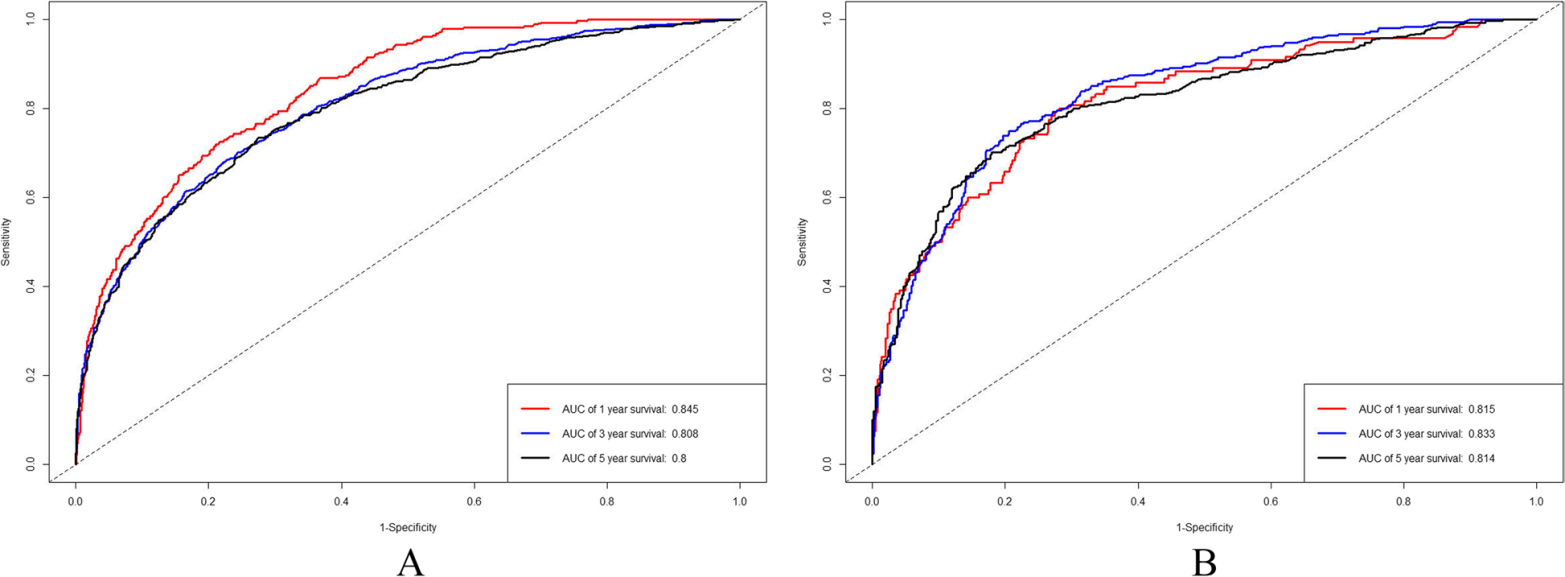
ROC curves of the nomogram for 1-year, 3-year and 5-year OS in development cohort (**A**) and validation cohort (**B**)

**Fig. 4:**
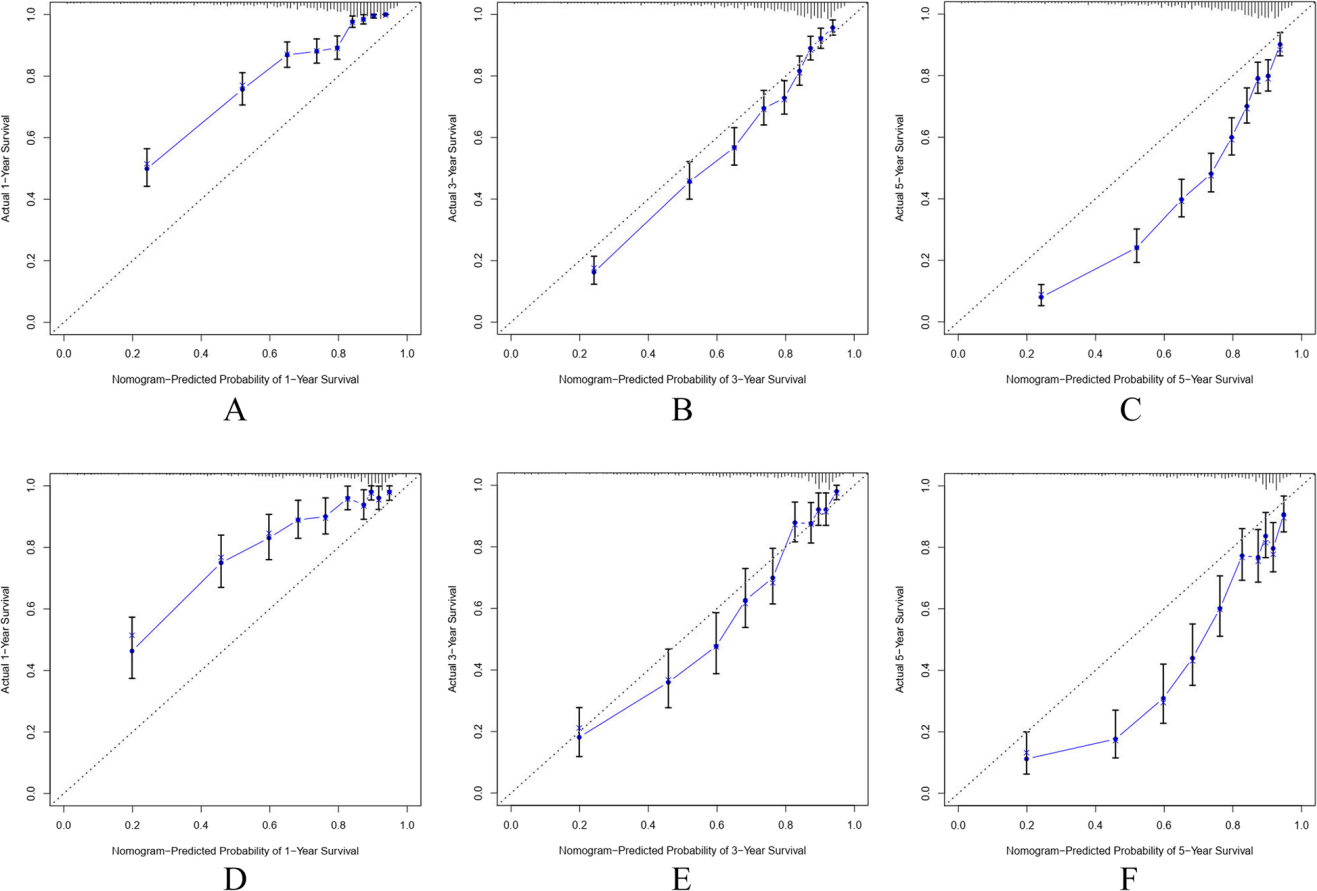
Calibration plots of development cohort for 1-year(**A**), 3-year(**B**) and 5-year(**C**) OS; calibration plots of validation cohort for 1-year(**D**),3-year(**E**) and 5-year (**F**) OS

**Fig. 5:**
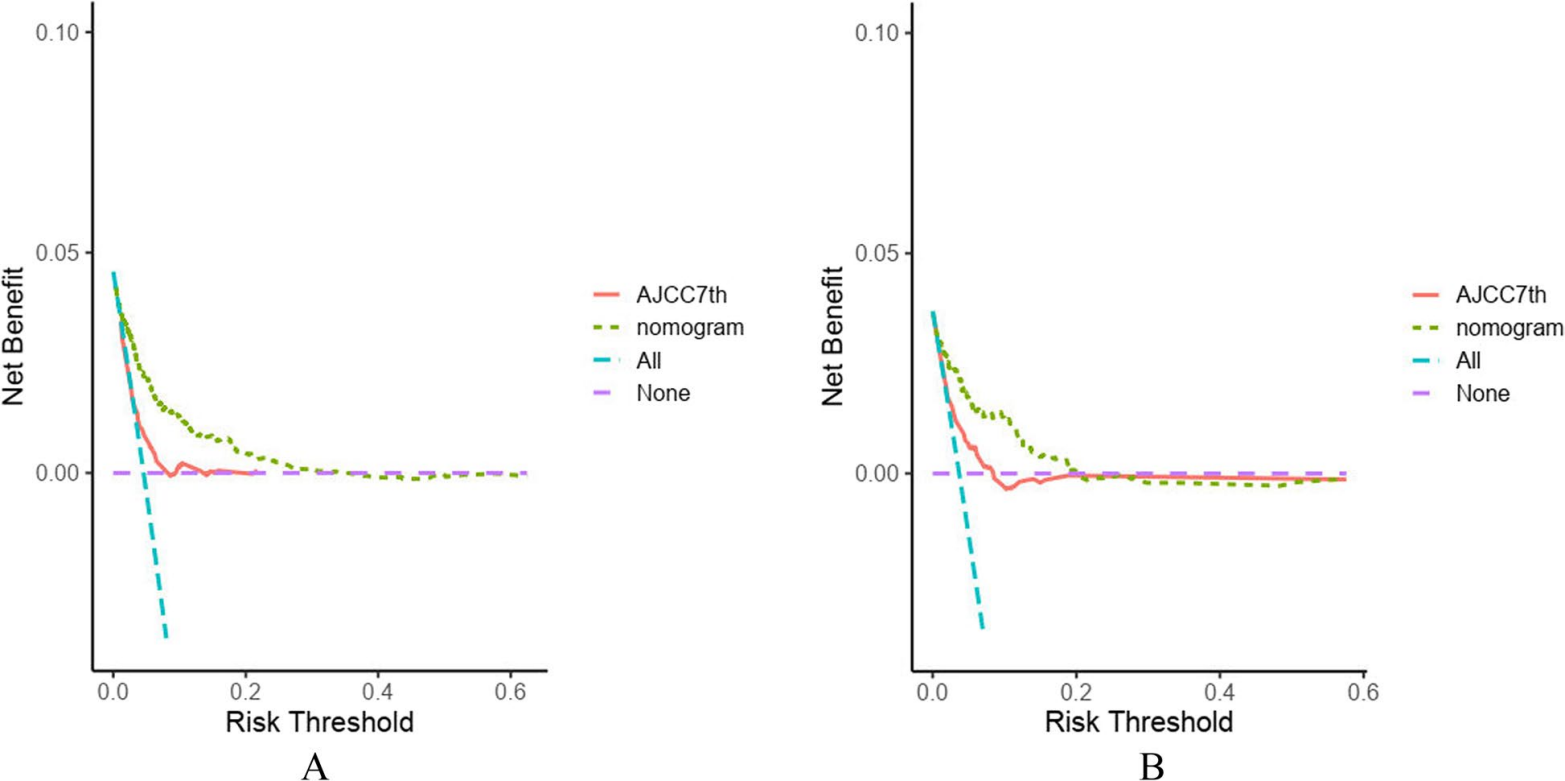
Decision curve analysis of nomogram and AJCC 7^th^ staging system for the survival prediction of PLN-RSJCs in development cohort(**A**) and validation cohort (**B**). (None: None of the patients have a bad outcome; All: Bad outcomes occur in all patients

In the development cohort, the calculated C-index of the generated nomogram for patient OS was 0.751 (0.737–0.765), which was more significant than that of the 7th AJCC stage 0.681 (0.665–0.697). Additionally, the performances of the nomogram were assessed by ROC curves, with AUC values of 0.845, 0.808 and 0.800 for 1-year, 3-year and 5-year OS, respectively (Fig. 3 A). Moreover, calibration plots for 1-year, 3-year and 5-year OS in the development cohort, confirmed the correlation between actual observations and predicted outcomes (Fig. 4 A-C). Decision curve analysis further showed that this nomogram prediction model performs better in terms of clinical prediction than of the 7th AJCC staging system (Fig. 5). In addition, an internal verification of the nomogram was performed in the validation cohort to evaluate its applicability. In this cohort, the calculated C-index was 0.750 (0.764–0.736), with AUC values of 0.815, 0.833 and 0.814 for 1-year, 3-year and 5-year OS, respectively (Fig. 3 B). As previously observed for the development cohort, the calibration curve confirmed the positive correlation between nomogram prediction and actual patient outcome (Fig. 4 D-F).

### Performance of the nomogram in patient of low risk group and high risk group

**Fig. 6:**
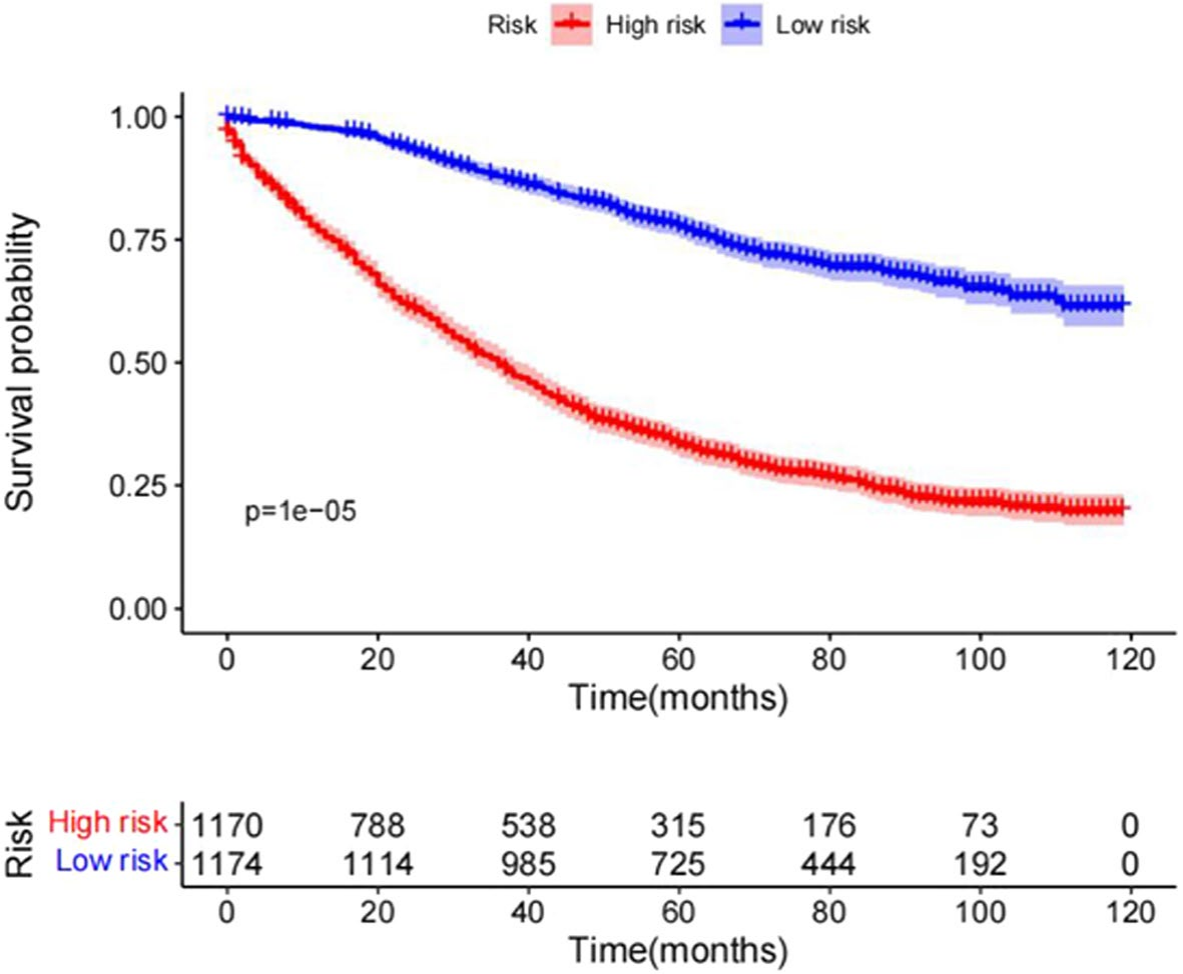
Kaplan–Meier overall survival curves of PLN-RSJCs with different risks stratified by the nomogram

The development cohort was stratified in two subgroups, according to the obtained score: low risk group: score < 94 points; high risk group: score ≥ 94 points. Kaplan–Meier survival curves showed a significant difference in OS between the two groups (*P* < 0.05; Fig. 6).

## Discussion

In ICD-O-3, the location codes for sigmoid colon, rectosigmoid junction and rectal cancers are, respectively, C18, C19 and C20, suggesting distinct anatomical tissues, and thus, different associated pathologies [14]. However, most clinical systems, such as the AJCC system, classify diseases of the rectosigmoid junction as rectal disease. A conclusive definition of RSJ is yet to be reached by the scientific and clinical community. However, it has been shown that RSJC is more prone to present lymphatic metastasis than cancer in the sigmoid and the rectum [15], and that the first is more likely to originate distant metastases than the latter [16]. Despite being from the same pathological subtype, rectosigmoid junction and rectal tumors are associated with different patient OS [15, 17], thus requiring distinct treatment modalities [18–20].

A nomogram risk prediction model for colorectal cancer patients has been developed, however lacking data on its pathological type and on the prognosis of lymph node positive patients [21–23]. Moreover, these reports have not distinguished RSJ from coloretal cancer [24, 25]. A study by B. Morcos et al*.* showed that the number of positive lymph nodes during surgery directly affects the choice of neoadjuvant chemoradiotherapy after colorectal cancer surgery [26]. Our study established a risk assessment of the presence of positive lymph nodes (PLN) in patients with RSJ adenocarcinoma after surgery. Age, marital, AJCC stage, tumor size, and regional lymph nodes exam were selected as independent risk predictors of PLN-RSJCs. Using these parameters, we validated the nomogram accuracy by C-index, AUC in the ROC, and calibration curves. To evaluate the clinical utility and potential benefits of the model, the decision curve analysis was applied to current study [27]. Overall, our results showed that our newly generated model performs better in the prediction of patient outcome than the currently used AJCC staging system.

Still, we acknowledge the limitations of the present study. First, given that the SEER database collects information from the American population, which are mostly Caucasian, the findings may be biased, even though we did not include race as a predictor in our model. Additionally, available data is incomplete namely for initial records. For example, immunohistochemistry results have only been recorded since 2010. Moreover, detailed therapeutic strategies for patients include chemotherapy plan and dose were not reported in the database. Radiation therapy also includes only radiotherapy sites and some techniques, such as seed implantation and external irradiation. Furthermore, we did not include any data on RSJC-associated tumor markers, such as CEA, Ca199, and Ca242. Finally, our study is limited by two important factors: the lack of analysis of cancer-specific survival (CSS), and the lack of external multi-center validation using patient cohorts in the clinical context. Therefore, additional research is advised to support our preliminary findings.

## Data Availability

If anyone wants to request the data from this study,please contact the author named Wu Yan long(wuyanlong578@126.com).
